# Comparative Morphological Effects of Cold-Blade, Electrosurgical, and Plasma Scalpels on Dog Skin

**DOI:** 10.3390/vetsci7010008

**Published:** 2020-01-12

**Authors:** Luca Lacitignola, Salvatore Desantis, Giovanni Izzo, Francesco Staffieri, Roberta Rossi, Leonardo Resta, Antonio Crovace

**Affiliations:** 1 Dipartimento dell’Emergenze e Trapianti di Organo, sez. Cliniche Veterinarie e Produzioni Animali, Università degli studi di Bari Aldo Moro, S.P. per Casamassima km.3, Valenzano, 70010 Bari, Italy; salvatore.desantis@uniba.it (S.D.); gioizzo@live.it (G.I.); francesco.staffieri@uniba.it (F.S.); antonio.crovace@uniba.it (A.C.); 2 Dipartimento dell’Emergenze e Trapianti di Organo, sez. Anatomia Patologica., Università degli studi di Bari Aldo Moro, Policlinico di Bari, Piazza G. Cesare 1, 70017 Bari, Italy; roberta.rossi@uniba.it (R.R.); leonardo.resta@uniba.it (L.R.)

**Keywords:** plasma scalpel, electrosurgery, thermal damage, scanning electron microscopy

## Abstract

The aim of the present study was to evaluate the histological results of the Onemytis^®^ plasma surgery device with Airplasma^®^ technology. We compared the efficacy and the effect on tissues of the new plasma electrocoagulation system with electrosurgery and a scalpel blade. Samples of healthy skin tissue from four dogs that underwent mastectomy were evaluated. Three different incision modes were used, i.e., a cold blade, electrosurgery, and the Onemytis^®^ plasma scalpel were evaluated histologically to assess invasiveness and tissue injuries at different distances from the cutting surface. The histological examinations showed moderate necrosis caused by Onemytis^®^, compared to the use of the more invasive electrosurgery, which induces thermal damage that extends beyond 1000 µm. Our study shows that the use of the plasma scalpel reduces the extension of the thermal lesion on the skin compared to an electrosurgical scalpel.

## 1. Introduction

The plasma scalpel is an electrosurgical device that uses pulsed radiofrequency to generate a plasma-mediated discharge along the exposed rim of an insulated blade. It is neither a laser nor a scalpel or radiofrequency device. Plasma, from the physical point of view, is believed to be the fourth state of aggregation of matter, between the liquid and the gaseous states, a sort of liquefied gas according to physicists. This plasma rim provides a cutting edge for precise tissue dissection with simultaneous hemostasis and lesser thermal damage by the blade at physiological body temperatures [1,2,3].

Airplasma^®^ is a newly developed technology that makes it possible to generate plasma energy directly from the air, therefore, without using other inert gases (Argon or Helium) [4,5,6]. The ionization process of the air is achieved through an electronic process. Initially neutral, the air is ionized by passing a strong high-frequency and high-voltage electromagnetic pulse through an electronic process. In this way, the insulating power of the air is eliminated, thus transforming it into an ideal conductor of energy. The generated plasma is visible in the form of a glow [4,5,6].

**Figure 1:**
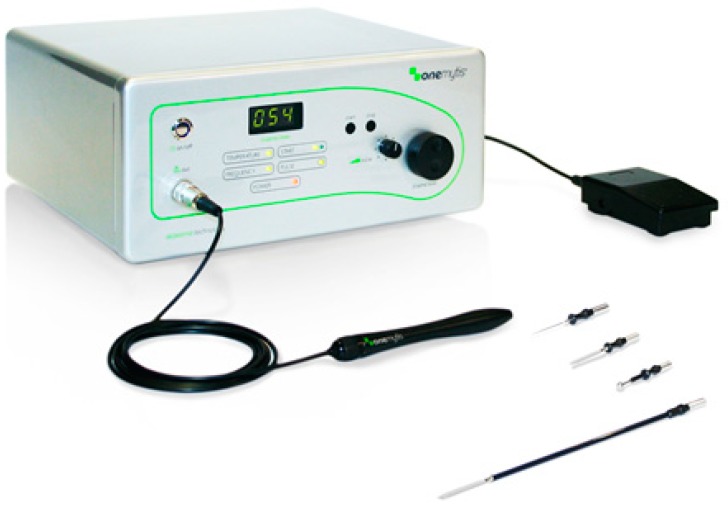
Onemytis^®^ device.

Onemytis^®^ is a plasma scalpel that uses Airplasma^®^ technology for a thermo-clotting-electro-plasma- device (Figure 1). According to the manufacturer, this device offers the following features: combined cutting, ablation, and coagulation function, reduces invasiveness due to the absence of return plates, necrotized area almost wholly absent or limited, operating temperature ≤50 °C, no need for protection for the operator and patient, and no use of inert gases.

Despite the increasing popularity and widespread use of the plasma scalpel, there is a paucity of data on morphological changes in injured tissues [7,8,9,10]. The aim of the present study was to compare the effects of the cold-blade, electrosurgical, and air plasma units on the skin morphology, using both light microscopy and scanning electron microscopy (SEM), also considering the type and spread (invasivity) of damage on the cut skin.

## 2. Materials and Methods

The study was performed on client-owned dogs presented to the veterinary teaching hospital, Department of Emergencies and Organ Transplantation of the University of Bari, Italy. Informed written consent was obtained for each patient before the inclusion in the study. All devices used in this study are approved for use in veterinary medicine in the United Europe.

The study included four female dogs, two mixed-breed and two Cocker Spaniel dogs, with an average age of 6.3 years and an average weight of 9.5 kg, diagnosed with mammary cancer and undergoing a total unilateral mastectomy. Premedication included acepromazine 0.002 mg/kg and morphine 0.3 mg/kg IM Anesthetic induction was performed with propofol 4–5 mg/kg IV. After endotracheal intubation, isoflurane in O_2_ was used for anesthetic maintenance. All patients were monitored during the recovery phase and received antibiotic, anti-inflammatory, and analgesic therapy.

### 2.1. Devices

As a plasma scalpel, the Onemytis^®^ device is constituted by a central unit equipped with intensity adjustment commands from 0 to 100, a sterilizable autoclave handpiece, a needle tip, and a delivery pedal (Figure 1).

Based on the manufacturer’s indications and a previously reported study [7], Onemytis^®^ was used with a medium–high intensity (about 70% of the maximum deliverable power) on skin tissue.

A scalpel blade number 22 was used for the skin and subcutaneous dieresis (cold blade). An electric scalpel, model SURTRON 160 in coagulation mode and power of 60, was employed as the electrosurgical unit.

### 2.2. Surgical Technique

Patients were placed in dorsal recumbency, and the sterile field was prepared. An elliptical incision of the skin and subcutaneous tissues was performed along the mammary chain with at least 3-cm margins of healthy tissue on each side.

The dieresis was performed with the cold blade for the thoracic with the electric scalpel for the abdominal, and with the plasma scalpel for the inguinal mammary chain. Once the exeresis of the surgical incision was completed, the three different portions were then prepared by isolating three normal skin and subcutaneous samples not affected by the neoplasia.

### 2.3. Histology Processing

Skin fragments of 1 cm^2^ were harvested and fixed in 4% (v/v) phosphate-buffered paraformaldehyde, pH 7.4, for 24 hours at 4 °C. After embedding in paraffin wax, serial sections (7-μm thick) were cut and stained with hematoxylin-eosin (Abcam, Cambridge, UK).

### 2.4. Scanning Electron Microscopy

Formalin-fixed skin fragments, after rinsing in phosphate buffer, were post-fixed in 1% OsO_4_ for 2 h at 4 °C, rinsed, dehydrated in an ethanol series, and then critical point dried using CO_2_. Specimens were mounted on stubs, coated with gold–palladium in a sputter coater, and examined using a Quanta 250 (FEI Company, Milan, Italy) scanning electron microscope (SEM).

### 2.5. Histomorphometric Analysis

To evaluate the presence, entity, and invasivity (width) of the thermal injury, stained sections at distances of 7, 77, 119, 300, 600, 700, 800, 900, and 1000 μm from the surgical incision were photographed with a 4´ lens using a light microscope (Eclipse Ni-U; Nikon, Tokyo, Japan) and analyzed with the image-analyzing program NIS Elements BR (Version 4.30) (Nikon, Tokyo, Japan).

For each slice, carbonization, coagulation, necrosis of the epithelium, detachment of the epidermis from the basement membrane, loss of cellular detail, cell fusion were considered to be alterations of the epidermis, whereas cellular hypereosinophilia, arrangement of bundles of collagen fibers, interstitial edema, hyperemia, lymphangiectasia were considered for the dermis. The following scores were assigned: 0 = normal; 1 = slight alteration; 2 = moderate alteration; 3 = severe alteration. 

The sum of the scores relative to the portions of the epidermis (Epidermal Score, ES), derma (Dermal Score, DS), and total (Total Score, TS) was then evaluated.

### 2.6. Statistical Analysis

Statistical analysis was performed using Medcalc software 14 (MedCalc Software bv, Ostend, Belgium). Semi-quantitative values were evaluated through the non-parametric Kruskal–Wallis test, and the mean and standard error are indicated. The significance was set at *p* < 0.05.

## 3. Results

### 3.1. Scanning Electron Microscopy

**Figure 2:**
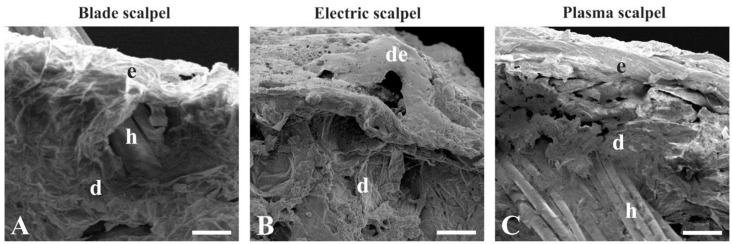
Scanning electron microscopy after skin incision using blade (**A**), electrosurgical (**B**), and plasma scalpels (**C**) in dogs undergoing total unilateral mastectomy. Note the higher condensation in skin removed by the electrosurgical and plasma scalpels compared to blade scalpel-excised skin. d, dermis; de, detached epidermis; e, epidermis; h, hair follicle. Scale bars: 100 μm.

Scanning electron microscopy analysis revealed different morphological features of the skin collected by the blade, electrosurgical, and plasma scalpels (Figure 2A–C). The skin isolated with the scalpel blade exhibited a thin epidermal layer overlying the dermis composed of loose connective tissue (Figure 2A). Skin excised with the electrosurgical unit was condensed in appearance and displayed detachment of the epidermis from the dermis, which showed a dense and irregular consistency (Figure 2B). Plasma scalpel-removed skin displayed a homogeneous condensation of both the epidermis and the derma (Figure 2C).

### 3.2. Light Microscopy

**Figure 3:**
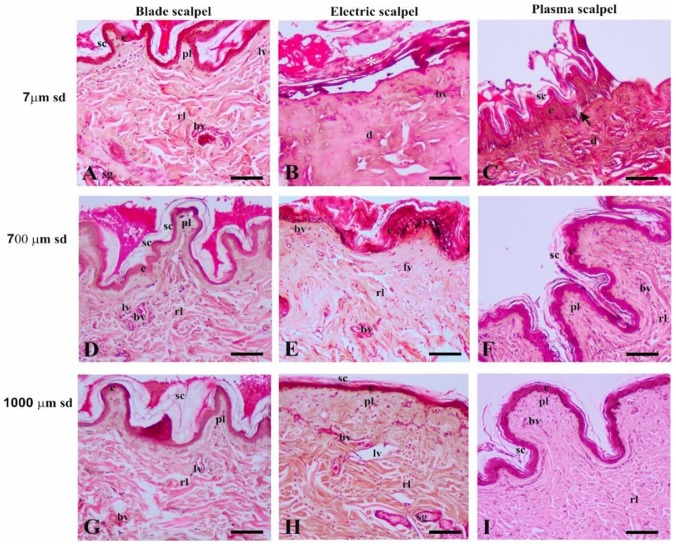
Histological features of canine skin after incision with blade scalpel (**A,D,G**), electrosurgical scalpel (**B,E,H**), and plasma scalpel (**C,F,I**) at 7 μm (A,B,C), 700 μm (D,E,F), and 1000 μm (G,H,I) from the surgical diaeresis. bv, blood vessels; e, epidermis; lv, lymphoid vessel; rl, reticular layer of the dermis; sb, sebaceous gland; sc, stratum corneum; sd, distance from the surgical diaeresis; pl, papillar layer of the dermis; *, detached epidermis; arrow, detaching epidermis. Scale bars: 100 μm.

Light microscopy analysis of the surgical incision showed a complete absence of carbonization and/or coagulation in tissue samples after cold-blade incisions, whereas different types and degrees of tissue damage were observed in the skin tissues after the electrosurgical unit or the Onemytis^®^ plasma scalpel. Figure 3 displays histological features of skin at three representative distances (7 µm, 700 µm, 1000 µm) and demonstrates the width of tissue injuries caused by surgical techniques.

#### 3.2.1. Distance of 7 µm from the Surgical Cutting Surface

The blade scalpel (Figure 3A) did not produce significant structural changes in the epidermis and dermis. In the latter, the blade induced the appearance of some highly eosinophilic collagen fibers in the reticular layer, which also displayed a mild edematous condition. Absence of tissue damage was also evidenced in the sweat and sebaceous glands. Blood and lymphoid vessels were intact.

The electrosurgical unit induced thermal stress carbonization and coagulative necrosis of the skin (Figure 3B). The epidermis showed focal detachment from the underlying structures as well as coagulation and detachment of the acidophilic stratum corneum. The dermis was homogeneously eosinophilic in aspect due to the presence of highly denatured and stretched collagen fibers, which appeared condensed and fused. The deep zone of the dermis exhibited clear spaces owing to the accumulation of moderate edema between collagen bundles. The connective tissue cells were poorly evident and strongly acidophilic in the papillary layer, with indistinguishable nuclei. The vascular structure was not detectable.

The Onemytis^®^ plasma scalpel (Figure 3C) caused coagulative necrosis of the skin. The epidermis showed focal signs of carbonization in the stratum corneum, highly eosinophilic keratinocytes in the most superficial zone of the stratum spinosum, and focal detachment of the stratum basale cells from the basement membrane. The coagulation necrosis in the dermis was significantly narrower than in the electrosurgical cut because this damage was homogeneously diffused in the papillary layer, whereas the reticular layer showed a mix of condensed and non-condensed highly eosinophilic collagen bundles. The latter were separated by diffuse edema. The vascular structures were not detectable.

#### 3.2.2. Distance of 700 µm from the Surgical Cutting Surface

Histological sections of skin at 700 µm and 7 µm from the scalpel blade incision (Figure 3D) were not significantly different.

At 700 µm from the electrosurgical incision, the skin (Figure 3E) displayed lesser damage than at the incision. The eosinophilic epidermis exhibited detachment of a condensed stratum corneum and coagulation necrosis of cell layers superjacent to the stratum basale, which contained features of focal carbonization. In the dermis, eosinophilic cells were scattered in a homogeneously dense connective tissue, which presented edema in the deeper zone. Signs of hyperemia and lymphangiectasia were also detected.

At this distance, the skin removed by the Onemytis^®^ plasma scalpel (Figure 3F) showed a slight detachment of the weakly eosinophilic stratum corneum from the unaltered epidermis. The dermis exhibited dense connective tissue in both the papillary and reticular layers. The scattered connective cells did not display injury signs. The deepest region of the dermis presented edema. Blood and lymphoid vessels were normally structured.

#### 3.2.3. Distance of 1000 µm from the Surgical Cutting Surface

At 1000 µm from the scalpel blade incision, the skin presented normal histomorphological features (Figure 3G).

At this distance, the skin removed with the electrosurgical unit (Figure 3H) exhibited a thin and weakly eosinophilic stratum corneum; the papillary layer of the dermis was thicker than in normal skin, whereas the reticular layer did not show significant damage to collagen fiber arrangement. The connective cells appeared normal in morphology. However, diffuse edema, hyperemia, and lymphangiectasia were detectable. The sebaceous glands appeared weakly eosinophilic.

The skin cut with the Onemytis^®^ plasma scalpel did not exhibit significant damage at 1000 µm away from the incision (Figure 3I), although the collagen bundles were more densely packed than in the skin removed with the two other types of blades. The vessels were not hyperemic, and there was no lymphangiectasia.

### 3.3. Histomorphological Damage Quantification

The related ES, DS, and TS are shown in Table 1, Table 2 and Table 3, respectively. The thermal damage caused by Onemytis^®^ extended laterally to the operative breach up to 700 μm in the epidermis portion, and 800 μm in the dermis portion. The extent of tissue damage at the same level was assessed as severe in incisions performed with electrosurgical units and with tissue lesions referable to thermal damage beyond 1000 μm from the surgical incision.

**Figure 4:**
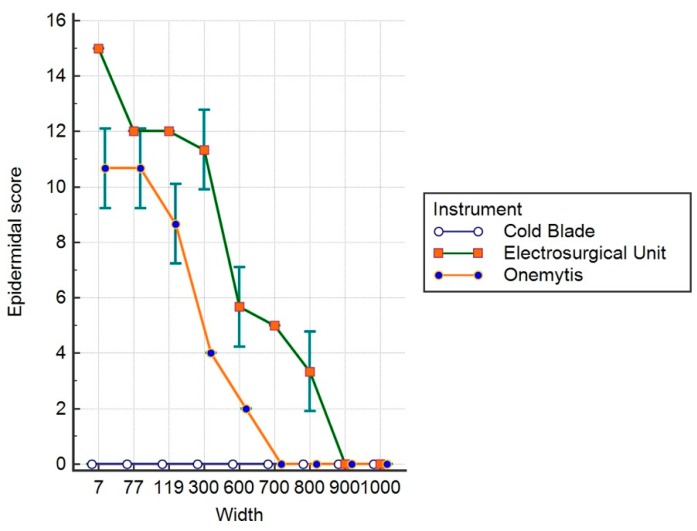
Epidermal score.

**Figure 5:**
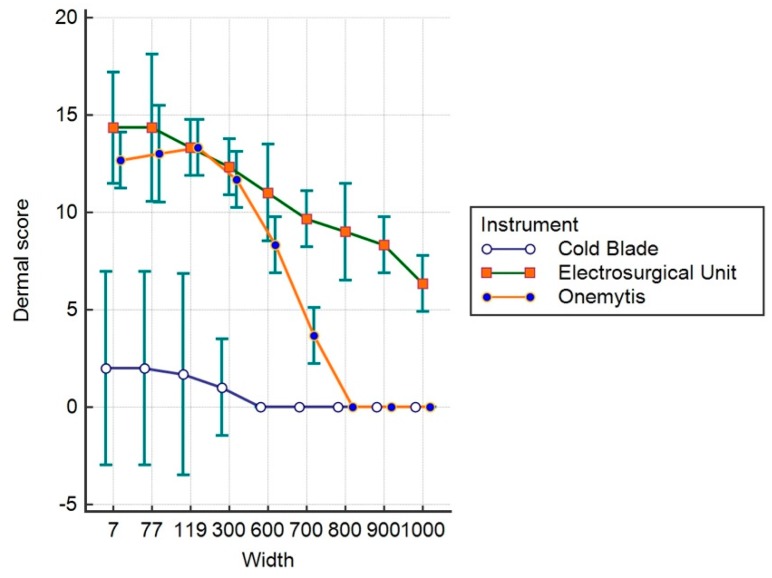
Dermal Score.

**Figure 6:**
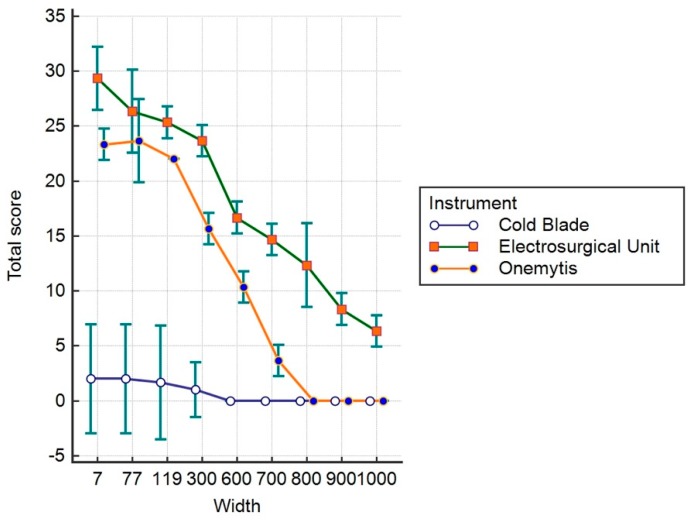
Total score.

Figure 4, Figure 5 and Figure 6 show the values relative to the ES, DS, and TS, respectively.

The data show that for the ES, the effects of the plasma and electric scalpels were significantly higher than the cold blade up to 600 μm (*p* < 0.05), with a greater difference for the electric scalpel compared to the plasma. The effects of the plasma scalpel were not different from the cold blade up to 700 μm (*p* > 0.05), while the effect of the electric scalpel was significantly higher up to 900 μm (*p* < 0.05).

Regarding the effects on the dermis (DS), both the electric scalpel and the plasma scalpel do not show differences in their effects up to 300 μm (*p* > 0.05), but they are still considerably more invasive than the cold blade. At 600 μm, the plasma scalpel is significantly less invasive than the electrical one (*p* < 0.05), overlapping the 800 μm cold blade; meanwhile, the electric scalpel is still significantly more invasive beyond 1000 μm (*p* < 0.05).

The sum of the effects on the epidermis and dermis evaluated with TS show significantly greater invasiveness of the electric scalpel compared to the cold blade and the plasma scalpel up to and over 1000 μm (*p* < 0.05). At 800 μm, no significant differences were observed between the plasma and the blade (*p* > 0.05).

## 4. Discussion

Plasma electrocoagulation allows effective cutting and coagulation without altering tissue healing, representing a significant innovation when compared with traditional techniques in surgery [7].

This study compared the effects of the cold blade, electrosurgical scalpel, and Onemytis^®^ plasma scalpel on normal skin, evaluating the histological aspects of thermal damage on the epidermis and dermis. Similar to previous findings, the plasma scalpel was superior to the electrosurgical unit in reducing tissue injury. Indeed, [7,8,9]. The histological examination of the skin biopsies showed an absence of necrosis of the epidermis and dermis with the use of the cold blade, moderate necrosis with the use of the Onemytis^®^ device, and severe necrosis with the use of the electrosurgical unit, as confirmed by the literature [7,8,9]. The thermal damage of skin tissues occurs from 0 to 600 μm adjacent to the surgical incision using the plasma scalpel, with a cellular resentment of damage up to 700 μm from the cutting edge. It is of considerable importance that the absence of carbonization and drying of the tissues leads to better and faster healing of the tissues than with electrosurgery. Tissue necrosis and the lateral thermal damage associated with the ischemia caused by the electrosurgical system may slow down tissue healing [7,8,9,10,11,12,13].

The technology on which Onemytis^®^ is based is the transformation of the air into an energy conductor thanks to the generation of high-voltage pulses through a high-frequency sinusoidal oscillator. In this way, the device can also operate without direct contact, using the air column interposed between the handpiece and the tip as an energy conductor, and not exceeding an average dissipation temperature of 50 °C [14]. In addition, the plasma scalpel confines its effect on the target tissue or vessel without carbonization, and with minimal thermal diffusion to adjacent tissues to reduce tissue damage.

The histological comparison concerning the lateral depth of the thermal damage induced by two devices, the plasma scalpel and the electrosurgical unit, showed a statistically significant difference between these two devices. In this study, we did not evaluate the deepness of the damage provided by the device. However, it has been reported that the plasma scalpel provides better control of surgical depth incision, limiting accidental damage to deep layers. [7,9,14]

Our current study confirms that high operating temperatures for deep thermal coagulation are not essential for tissue hemostasis as used with electrosurgery. For example, the plasma scalpel uses lower temperatures and showed reduced thermal damage to adjacent tissues.

A major limitation of this study is the lack of wound healing assessment. This was not feasible due to the nature of our client-owned canine population recruited for the study. However, some studies on pig skin have demonstrated that the histologic scoring for injury and wound strength was equivalent between the plasma scalpel and cold blade incisions after the 6th week [8]. The latter device promoted better healing both in canine species and rats [4,8,9]. Moreover, it has been demonstrated that the use of a plasma scalpel on human skin produces better cosmetic outcomes compared with electrosurgery, because of the reduced duty cycle that allows for efficient cooling of the plasma blade. [1,8]

This study aimed to assess the histological effects of thermal injury and coagulative necrosis produced by a plasma scalpel compared with other techniques. Hemostasis was not considered, and it should be considered another limitation of the study. Other studies confirmed that the plasma scalpel produces proper bleeding control in animals and humans [7,8]. In particular, Loh et al. demonstrated that the plasma scalpel significantly reduces bleeding compared with the scalpel and is comparable to traditional electrosurgical devices [8]. It has been supposed that the coagulative necrosis was able to control the bleeding by a combination of the denaturation of proteins and molecules, tissue shrinkage, and vessel sealing due to the fusion of blood vessel collagen and elastic fibers. Another possible mechanism of hemostasis produced by the plasma scalpel may be a nonthermal vasoconstrictive and thrombotic effect [15]. In our study, the plasma scalpel produced coagulative necrosis effects with less width tissue damage resulting in a more precise tissue dissection. This could be due to lower blade temperature than electrosurgery, but further studies are warranted to compare these techniques in terms of hemostasis. We demonstrated that the plasma scalpel provides an efficient skin surgical incision comparable to that of the cold-blade scalpel. These results make Onemytis^®^ a potential device in various surgical fields where electrosurgery is not used extensively for fear of tissue damage.

The Onemytis^®^ plasma scalpel has already been used in human and veterinary medicine with several publications [7,8,9,11], and the results of our study suggest that Onemytis^®^ provides useful advantages over conventional electrosurgery.

## 5. Conclusions

We demonstrated that the plasma scalpel provides efficient skin incision with a superior wound profile, comparable to that of the cold blade but with significantly less bleeding, and lower thermal damage compared to electrosurgery. These results suggest that the plasma scalpel has an interesting potential in surgical fields where electrosurgery is not used extensively.

## Figures and Tables

**Table 1:** Epidermal Score.

Device	Mean	Std. Error	95% Confidence Interval
**Cold Blade**	2.53	0.05238	−0.1050 to 0.1050
**Electrosurgical Unit**	7.1481	0.05238	7.0431 to 7.2532
**Onemytis^®^**	4	0.05238	3.8950 to 4.1050

**Table 2:** Dermal Score.

Device	Mean	Std. Error	95% Confidence Interval
**Cold Blade**	0.7407	0.1789	0.3821 to 1.0994
**Electrosurgical Unit**	1.,963	0.1789	10.6043 to 11.3216
**Onemytis^®^**	6.963	0.1789	6.6043 to 7.3216

**Table 3:** Total Score.

Device	Mean	Std. Error	95% Confidence 3nterval
**Cold Blade**	0.7407	0.1852	0.3695 to 1.1120
**Electrosurgical Unit**	18.1111	0.1852	17.7398 to 18.4824
**Onemytis^®^**	10.963	0.1852	10.5917 to 11.3342
